# Pneumomediastinum as a complication of robotic-assisted radical nephrectomy: A case report

**DOI:** 10.14440/bladder.0194

**Published:** 2025-10-06

**Authors:** Ahmed Nafie, Ayman Agag, Abhishek Reekhaye, Muddassar Hussain, Manar Malki, Neil Barber

**Affiliations:** Department of Urology, Frimley Park Hospital, Camberley, Surrey GU167 UJ, United Kingdom

**Keywords:** Radical nephrectomy, Esophageal, Pneumomediastinum

## Abstract

**Background:**

Pneumomediastinum represents a rare but potentially serious complication of robotic-assisted radical nephrectomy, often resulting from carbon dioxide insufflation during retroperitoneal surgery.

**Case presentation:**

Reported here is a case of a 78-year-old female who underwent an uneventful robotic-assisted radical nephrectomy for a right renal mass but presented 5 days postoperatively with shortness of breath and chest discomfort. Imaging confirmed extensive pneumomediastinum without esophageal perforation. She was managed conservatively with oxygen therapy, pain control, and a soft diet, leading to full recovery.

**Conclusion:**

Awareness of risk factors, such as a low body mass index, prolonged operative time, and extensive retroperitoneal dissection, is essential for early recognition and management of this rare complication.

## 1. Background

Robotic-assisted surgery has emerged as a significant surgical innovation, offering enhanced surgical practices that include better visualization, a wider range of mechanical movements, enhanced ergonomic efficiency, and improved overall results.^1^ This approach is particularly useful for procedures that necessitate entry into the retroperitoneal space. However, complications associated with robotic surgery have been reported, such as hypercarbia, pneumothorax, and gas embolism.^2^,^3^ The presence of free air inside the mediastinal space is defined as pneumomediastinum, which may lead to serious complications, including cardiac collapse and death if not treated ideally.^4^

## 2. Case presentation

A 78-year-old woman was incidentally found to have a 4.2-cm mass arising from the anterior aspect of the right kidney during evaluation for abdominal pain, vomiting, and constipation. She underwent full staging and review by the regional renal cancer specialist multidisciplinary team, which determined stage T1bN0M0 disease. She was otherwise fit and well, with a past surgical history of hysterectomy and shoulder surgery.

She began to complain of severe back pain related to a T12 compression fracture and osteoporosis. Her renal function was normal, and she was offered a right robot-assisted radical nephrectomy. She was 157 cm tall and weighed 55 kg, with an estimated body mass index (BMI) of 22.31 kg/m^2^. The pre-operative physical examination was unremarkable.

She underwent a right robot-assisted radical nephrectomy in March 2024. Direct laryngoscopy showed a Cormack-Lehane grade IIb view. Endotracheal intubation with a video laryngoscope was challenging due to jaw limitations. The patient was placed in the left lateral position with a table break. A four-port retroperitoneal approach was performed with the Davinci system docked, and the peak end-tidal carbon dioxide (CO_2_) (PETCO_2_) was maintained at 40 mm Hg. CO_2_ insufflation pressure ranged from 12 to 14 mmHg and was adjusted as required.

During the operation, Gerota’s fascia was incised, and the right ureter was identified and traced to the hilum. A single renal artery and vein were identified and secured with hemo-lock clips. The right kidney was mobilized, and the adrenal gland was separated. The kidney was removed with an estimated blood loss of 20 mL. The operation was uneventful. The post-operative period was similarly uneventful, and the patient was discharged after 48 h. However, she re-presented to the emergency department 5 days later with shortness of breath, central chest pain, and sensation of tightness without nausea, vomiting, cough, or fever.

**Figure 1:**
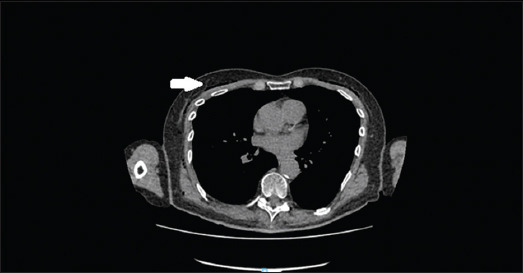
Several small pockets of gas (indicated by the white arrow) were demonstrated adjacent to the superior portion of the esophagus (abdominal window)

**Figure 2:**
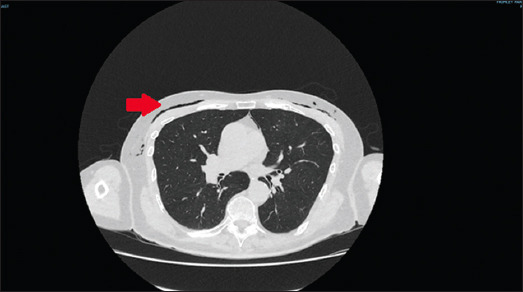
Several small pockets of gas (indicated by the red arrow) were demonstrated adjacent to the superior portion of the esophagus (lung window)

On examination, there was surgical emphysema on the upper chest bilaterally, which was non-tender. Computed tomography of the neck and thorax with oral contrast showed no evidence of esophageal perforation but revealed extensive pneumomediastinum and subcutaneous emphysema (Figures 1 and 2).

A trace of fluid was noted in the right upper abdomen, consistent with recent surgery. The electrocardiogram and troponin levels were normal. She was managed conservatively with oxygen therapy, pain killers, and a soft diet, and was discharged after 48 h. The histopathology revealed an oncocytoma (benign), and the patient was subsequently discharged from the renal cancer service.

## 3. Discussion

Pneumomediastinum is generally considered a benign condition with a good prognosis in most cases. Serious underlying pathology should be ruled out, and management primarily focuses on relieving symptoms. Patients are typically admitted for at least 1 day for observation. Physical activity should be limited, and bed rest is recommended. Pain management, anxiolytic medications may be administered according to local protocols. Cough suppression with mild antitussives is advised, and supplemental oxygen can be used, as it may accelerate gas resorption.^5^-^8^ It is important to appropriately manage any pre-existing conditions, such as asthma or chronic obstructive pulmonary disease. Many studies suggest that discharge criteria include adequate pain control, stability of the pneumomediastinum, and resolution of potential complications such as pneumothorax.^7^ A rare sequel of pneumomediastinum is massive mediastinal air accumulation, usually resulting from missed esophageal or pulmonary injury with significant air leakage.^2^ In these cases, although rare, the simple pneumomediastinum may progress to a malignant one, leading to tamponade and airway compression. In such cases, video-assisted thoracoscopic surgery or even thoracotomy may be performed emergently for mediastinal decompression.^4^,^6^

A robotic retroperitoneal approach in radical or partial nephrectomy, as well as nephroureterectomy, has numerous benefits over the traditional transperitoneal surgical approach. This includes direct access to the renal hilum and posterior tumors, and avoidance of the peritoneal cavity, especially in patients with a hostile abdomen.^9^ Over the past decade, retroperitoneal surgery has been increasingly utilized, though CO_2_ insufflation may occasionally cause subcutaneous emphysema and, rarely, pneumomediastinum by forming subcutaneous space tracks.^10^,^11^

Despite being rare, pneumomediastinum can develop into a potentially fatal emergency due to hemodynamic compromise. In robotic surgery, a retroperitoneal approach is linked to an increased risk of subcutaneous emphysema developing into pneumomediastinum.^12^ Pneumomediastinum and even pneumopericardium are uncommon but potentially dangerous complications that can arise from retroperitoneal gas entering the mediastinum through the diaphragmatic hiatus due to the lack of subdiaphragmatic peritoneum.^13^

Tension pneumomediastinum can occur in rare instances where a significant volume of free air enters the mediastinum. The pressure from this air may affect venous return and have a direct effect on the heart. Thoracic decompression might be required in severe cases.^14^ In contrast, conservative measures, such as pain management and oxygen therapy, are typically used when pneumomediastinum is identified as a consequence of robotic surgery.

In the presenting case, potential risk factors included a low BMI, a relatively long operative time of approximately 90 min, the use of four robotic ports, extensive renal dissection to address the lesion, and a history of hysterectomy, which contributed to adhesions and increased operative difficulty and complication risk. Low BMI, extended operating duration, many surgical ports, and advanced age have been identified as risk factors for the development of subcutaneous emphysema as a consequence of robot-assisted surgery.^15^ Steep Trendelenburg posture and the necessity of substantial retroperitoneal resection were further factors that probably elevated the risk. The anesthesiologist should be on the lookout for signs and symptoms of subcutaneous emphysema in these situations, such as increasing crepitus and hypercarbia.^16^

The delayed presentation of this case may be related to occult micro-perforations that occur during mobilization, allowing the gradual tracking of CO_2_ along fascial planes with late decompression. Cough/Valsalva can also cause alveolar rupture (Macklin effect).^17^-^19^ In elderly patients, tissue fragility and slower healing plausibly may prolong small leaks and delay presentation and symptoms.

Quick action should be taken after diagnosis to prevent the condition from deteriorating, such as lowering insufflation pressure to achieve elimination of pneumoperitoneum. To prevent further complications, subcutaneous emphysema and the uncommon complication of pneumomediastinum must be diagnosed early, treated immediately, and monitored closely.^20^ Early recognition of risk factors is essential. These include the use of ≥4 trocars, loose or slipping trocars, protracted operative time, and elevated insufflation pressure. Such risks can be minimized through careful pre-operative planning, vigilant intraoperative monitoring, and meticulous surgical technique with suitable CO_2_ pressure.

## 4. Conclusion

Pneumomediastinum is a rare but important complication of robotic-assisted retroperitoneal nephrectomy, likely related to CO_2_ insufflation and patient-specific risk factors. Although typically self-limiting and managed conservatively, early recognition and appropriate supportive care are essential to prevent serious outcomes.

## Data Availability

No datasets were generated or analyzed during the current study.
